# Exclusive Breastfeeding Duration and Risk of Childhood Cancers

**DOI:** 10.1001/jamanetworkopen.2024.3115

**Published:** 2024-03-26

**Authors:** Signe Holst Søegaard, Mie Mølgaard Andersen, Klaus Rostgaard, Olafur Birgir Davidsson, Sjurdur Frodi Olsen, Kjeld Schmiegelow, Henrik Hjalgrim

**Affiliations:** 1 Danish Cancer Institute, Danish Cancer Society, Copenhagen, Denmark; 2 Department of Epidemiology Research, Statens Serum Institut, Copenhagen, Denmark; 3 Department of Pediatrics and Adolescent Medicine, University Hospital Rigshospitalet, Copenhagen, Denmark; 4 Department of Public Health, University of Copenhagen, Copenhagen, Denmark; 5 Harvard T.H. Chan School of Public Health, Boston, Massachusetts; 6 University of the Faroe Islands, Torshavn, Faroe Islands; 7 Institute of Clinical Medicine, Faculty of Medicine, University of Copenhagen, Copenhagen, Denmark; 8 Department of Hematology, University Hospital Rigshospitalet, Copenhagen, Denmark

## Abstract

**Importance:**

Breastfeeding has been suggested to protect against childhood cancers, particularly acute lymphoblastic leukemia (ALL). However, the evidence stems from case-control studies alone.

**Objective:**

To investigate whether longer duration of exclusive breastfeeding is associated with decreased risk of childhood ALL and other childhood cancers.

**Design, Setting, and Participants:**

This population-based cohort study used administrative data on exclusive breastfeeding duration from the Danish National Child Health Register. All children born in Denmark between January 2005 and December 2018 with available information on duration of exclusive breastfeeding were included. Children were followed up from age 1 year until childhood cancer diagnosis, loss to follow-up or emigration, death, age 15 years, or December 31, 2020. Data were analyzed from March to October 2023.

**Exposure:**

Duration of exclusive breastfeeding in infancy.

**Main Outcomes and Measures:**

Associations between duration of exclusive breastfeeding and risk of childhood cancer overall and by subtypes were estimated as adjusted hazard ratios (AHRs) with 95% CIs using stratified Cox proportional hazards regression models.

**Results:**

A total of 309 473 children were included (51.3% boys). During 1 679 635 person-years of follow-up, 332 children (0.1%) were diagnosed with cancer at ages 1 to 14 years (mean [SD] age at diagnosis, 4.24 [2.67] years; 194 boys [58.4%]). Of these, 124 (37.3%) were diagnosed with hematologic cancers (81 [65.3%] were ALL, 74 [91.4%] of which were B-cell precursor [BCP] ALL), 44 (13.3%) with central nervous system tumors, 80 (24.1%) with solid tumors, and 84 (25.3%) with other and unspecified malignant neoplasms. Compared with exclusive breastfeeding duration of less than 3 months, exclusive breastfeeding for 3 months or longer was associated with a decreased risk of hematologic cancers (AHR, 0.66; 95% CI, 0.46-0.95), which was largely attributable to decreased risk of BCP-ALL (AHR, 0.62; 95% CI, 0.39-0.99), but not with risk of central nervous system tumors (AHR, 0.96; 95% CI, 0.51-1.88) or solid tumors (AHR, 0.87; 95% CI, 0.55-1.41).

**Conclusions and Relevance:**

In this cohort study, longer duration of exclusive breastfeeding was associated with reduced risk of childhood BCP-ALL, corroborating results of previous case-control investigations in this field. To inform future preemptive interventions, continued research should focus on the potential biologic mechanisms underlying the observed association.

## Key Points

**Question:**

Is longer duration of exclusive breastfeeding associated with decreased risk of childhood cancers, including acute lymphoblastic leukemia (ALL), the most common cancer in childhood?

**Findings:**

In this cohort study including 309 473 Danish children, exclusive breastfeeding for at least 3 months was associated with decreased risk of childhood hematologic cancers, particularly B-cell precursor ALL, but not with risk of central nervous system or solid tumors.

**Meaning:**

Longer breastfeeding duration may be a potential factor in prevention of childhood B-cell precursor ALL.

## Teaser

This cohort study investigates whether the duration of exclusive breastfeeding is associated with risk of developing acute lymphoblastic leukemia and other childhood cancers.

## Introduction

In Europe, cancer is diagnosed in 1 in 350 children before age 15 years and is the leading disease-related cause of death in childhood after infancy.^1,2^ At least 10% of cancers in childhood are attributable to rare germline mutations,^3,4^ yet the etiology of most childhood cancers remains obscure.^5^ Consequently, there are currently no established preventive measures.

In this void, emerging research suggests that breastfeeding is associated with reduced risk of childhood cancers, such as acute lymphoblastic leukemia (ALL), the most common cancer in childhood. Meta-analyses and pooled studies have shown that children breastfed for at least 6 months had an approximately 20% lower risk of developing ALL or leukemia in general compared with those breastfed for shorter durations or not at all.^6,7,8,9,10,11^ Additionally, meta-analyses considering various durations of breastfeeding have found associations of breastfeeding with reduced risks of childhood acute myeloid leukemia (AML),^6,7,11^ Hodgkin lymphoma, and neuroblastoma.^7,10^

That breastfeeding may offer protection against childhood ALL is not without biologic credence given the pivotal role of breastfeeding in the shaping of the infant gut microbiome and immune system.^12,13,14^ In line with this, aberrant immune responses to infectious stimuli are believed to play a central role in the development of B-cell precursor (BCP) ALL, the most common ALL subtype, in childhood.^15,16^ Studies using murine models have reported direct links between the gut microbiome and BCP-ALL pathogenesis, indicating that undisturbed, complex, and species-rich microbiomes protect against BCP-ALL development.^16,17,18^

The suggested protective effect of breastfeeding against childhood cancer is noteworthy. It not only points to potential biologic pathways that could modulate childhood cancer risk but also suggests a simple preventive measure. It is therefore crucial to revisit and corroborate previous observations of reduced childhood cancer risk in breastfed children,^6,7,8,9,10,11^ as existing evidence stems from case-control studies, which are inherently vulnerable to recall and selection biases.

In this study, we leveraged the administrative records from the Danish National Child Health Register (DNCHR) to conduct a register-based cohort study. We aimed to investigate the association between exclusive breastfeeding duration and risk of childhood cancers among children born in Denmark between 2005 and 2018.

## Methods

According to the European Union General Data Protection Regulation (Article 30), this cohort study was listed in the record of processing activities for research projects in and was approved by the Danish Cancer Society. As per Danish law, purely register-based studies such as the present one do not require ethics approval or informed consent. The study followed the Strengthening the Reporting of Observational Studies in Epidemiology (STROBE) reporting guideline for cohort studies.

We identified all children born in Denmark from January 2005 to December 2018 in the Danish Civil Registration System.^19^ For each child, the information retrieved from the register included sex, date of birth, vital status (ie, dates of death or emigration), residence, birth dates of parents and siblings, and parents’ place of birth. Furthermore, using the unique identification number issued to all individuals living in Denmark as the key, we linked with the Medical Birth Register to obtain information on birth characteristics and with the Population Education Register to ascertain the mother’s highest educational level.^20,21^ Children missing information on birth weight, gestational age, mother’s age, and mother’s educational level were excluded from the analyses. We further excluded children with Down syndrome (*International Statistical Classification of Diseases and Related Health Problems, Tenth Revision* code Q90) through linkage with the Danish National Patient Register due to their increased risk of leukemia with distinct biology.^22,23^

Subsequently, we linked the cohort with the DNCHR to obtain information on breastfeeding.^24^ This database holds information collected by health care nurses at regular home visits during the child’s first years of life. These visits are offered to all parents of newborns in Denmark to monitor the child’s health and to provide guidance to parents regarding their child’s development, feeding, and other health-related factors.

In the database, breastfeeding data specifically pertain to the duration of exclusive breastfeeding. This period was defined as when lactation was the child’s primary nutrition supplemented only by water and/or, at most, 1 formula milk meal weekly.^25^ We extracted dates of exclusive breastfeeding cessation from the database, that is, when the child first received multiple formula meals weekly or solid foods.

Between 2005 and 2011, only a select number of Danish municipalities used the DNCHR. Beginning in 2012, reporting to the database became mandatory for all municipalities, and most complied. Accordingly, we excluded children from the cohort if their breastfeeding information was missing in the database. We calculated exclusive breastfeeding duration as the time from birth to the date of exclusive breastfeeding cessation. We classified children as “never breastfed” if the date of exclusive breastfeeding cessation was less than 14 days after birth, assuming that the cumulative exposure to breastfeeding was minimal among these children. In addition, we identified children diagnosed with cancer at ages 1 to 14 years through linkage with the Danish Cancer Register^26^ using morphology and topography codes defined in the *International Classification of Childhood Cancer* (Third Edition) (*ICCC-3*).^27^

### Statistical Analysis

The children in the cohort were followed up from age 1 year until the date of childhood cancer diagnosis, loss to follow-up or emigration, death, age 15 years, or December 31, 2020, whichever occurred first. We used Cox proportional hazards regression models stratified by sex and birth order (1, 2, or ≥3) with age as the underlying time scale to estimate hazard ratios (HRs) and 95% CIs for the association between exclusive breastfeeding duration and childhood cancer risk. Analyses were conducted from March to October 2023 using R, version 4.2.2 (R Project for Statistical Computing) on a dedicated Statistics Denmark server. *P* values and 95% CIs were derived using likelihood ratios. Two-sided *P* < .05 was considered significant.

Based on their potential association with both breastfeeding duration and childhood cancer risk, we adjusted for several potential confounders: mother’s age at delivery (linearly, 1-year intervals), birth weight (linearly, 1-g intervals), gestational age (linearly, 1-day intervals), mode of birth (vaginal or cesarean delivery), birth year (linearly), and mother’s highest achieved educational level (low: mandatory school, ≤9 years; medium: upper secondary or high school or vocational education, 10-12 years; and high: ≥13 years). Analyses were carried out for childhood cancers combined and for the 3 major *ICCC-3* subtypes: hematologic cancers, central nervous system (CNS) tumors, and solid tumors. Additionally, we conducted separate analyses for ALL and BCP-ALL (morphology code 9835) and neuroblastoma (morphology codes 9490 and 9500).

In analyses of childhood hematologic cancer, ALL, and BCP-ALL, we divided the duration of exclusive breastfeeding into approximately 3-month intervals: never or less than 14 days, 14 days to 2 months, 3 to 5 months, and 6 or more months. Additionally, to increase statistical power, breastfeeding duration was grouped into 2 categories (0-2 months and ≥3 months) to compare longer and shorter breastfeeding durations while also considering the number of events. For CNS and solid tumors, we analyzed exclusive breastfeeding duration in 2 categories only (0-2 months and ≥3 months) due to few events. For childhood cancers combined and for the aforementioned cancer subtypes, we also assessed log-linear trends in HRs for the association with exclusive breastfeeding duration per month excluding children who were exclusively breastfed never or for less than 14 days.

In supplementary analyses, we assessed the risk of childhood BCP-ALL associated with exclusive breastfeeding duration according to birth cohort with voluntary vs mandatory reporting to the DNCHR (2005-2011 vs 2012-2018), the attained age at which BCP-ALL incidence peaks (2-6 years) vs older ages (7-14 years), and birth mode (vaginal vs cesarean delivery). The proportional hazards assumption was evaluated by visual inspection of scaled Schoenfeld residuals. No obvious violations were detected.

## Results

Information on duration of exclusive breastfeeding was available for 318 034 children. This represented 44.9% of the 708 521 children born between 2005 and 2018 in municipalities that contributed to the DNCHR. Database coverage varied over time, from 63 066 of 296 871 children (21.2%) born between 2005 and 2011, when reporting was voluntary, to 254 968 of 411 650 children (61.9%) born between 2012 and 2018, when reporting was mandatory. The Figure provides an overview of inclusion in the study, with criteria for exclusion, case numbers, and reasons for censoring. The final study cohort included 309 473 children (51.3% boys; 48.7% girls).

**Figure.**
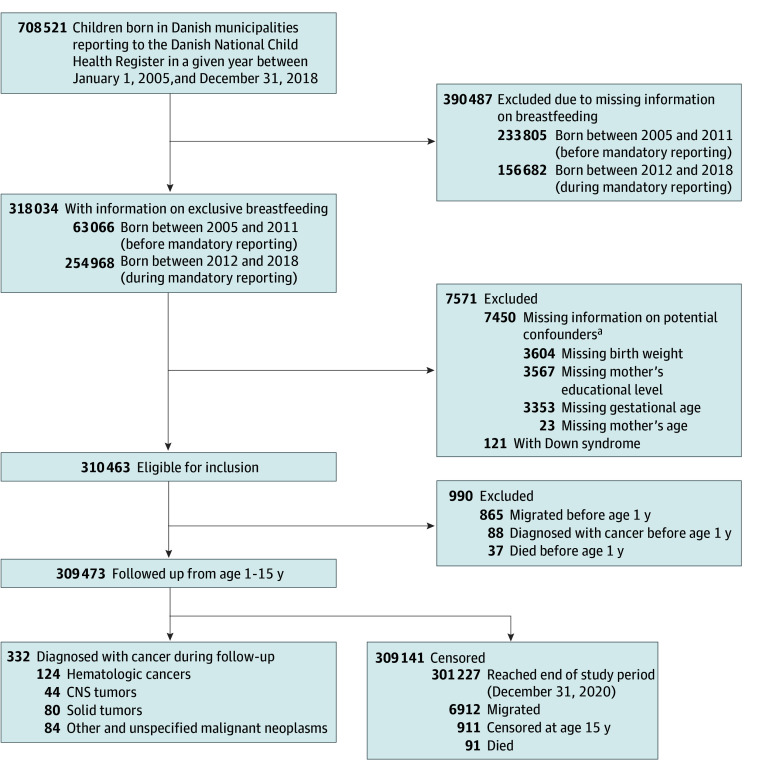
Flowchart of Inclusion in the Study CNS indicates central nervous system. ^a^Some individuals were missing information in more than 1 category.

During 1 679 635 person-years of follow-up, a total of 332 children (0.1%) were diagnosed with cancer (mean [SD] age at diagnosis, 4.24 [2.67] years; 194 boys [58.4%]; 138 girls [41.6%]); 124 (37.3%) had hematologic cancers, 44 (13.3%) had CNS tumors, 80 (24.1%) had solid tumors, and 84 (25.3%) had other and unspecified malignant neoplasms. Among the children diagnosed with hematologic cancers, 81 (65.3%) had ALL, of whom 74 (91.4%) had BCP-ALL; 7 (5.6%) were diagnosed with AML, and fewer than 5 were diagnosed with Hodgkin lymphoma. The most frequently diagnosed CNS tumors were astrocytoma, ependymoma, and intracranial and intraspinal embryonal tumors. Kidney tumors, neuroblastoma, and soft tissue sarcomas were the most common solid tumors (eTable 1 in Supplement 1).

Compared with the entire cohort, children diagnosed with cancer were less likely to be the first born in their families and to have a higher weight at birth. In addition, children with ALL were more likely to have a mother aged 35 years or older at the time of delivery compared with children in the entire cohort (Table 1).

**Table 1.** Baseline Characteristics of the Study Population and Duration of Exclusive Breastfeeding in Infancy According to Childhood Cancer Overall and ALL

Characteristic	Children, No. (%)		
	All (N = 309 473)	With any cancer (n = 332)	With ALL (n = 81)
Sex			
Boys	158 613 (51.3)	194 (58.4)	50 (61.7)
Girls	150 860 (48.7)	138 (41.6)	31 (38.3)
Birth order			
1	150 452 (48.6)	141 (42.5)	34 (42.0)
2	111 651 (36.1)	129 (38.8)	30 (37.0)
≥3	47 370 (15.3)	62 (18.7)	17 (21.0)
Birth weight, g			
<2500	13 349 (4.3)	13 (3.9)	<5^a^
2500 to <3000	36 143 (11.7)	37 (11.2)	<10^a^
3000 to <3500	101 762 (32.9)	102 (30.7)	29 (35.8)
3500 to <4000	107 187 (34.6)	105 (31.6)	21 (26.0)
≥4000	51 032 (16.5)	75 (22.6)	22 (27.2)
Gestational age, wk			
<36	10 053 (3.2)	9 (2.7)	<5^a^
36-37	21 929 (7.1)	35 (10.6)	<15^a^
38-39	108 327 (35.0)	100 (30.1)	32 (39.5)
40-41	160 929 (52.0)	178 (53.6)	35 (43.2)
≥42	8235 (2.7)	10 (3.0)	<5^a^
Mode of birth			
Vaginal	246 497 (79.7)	269 (81.0)	66 (81.5)
Cesarean delivery	62 976 (20.3)	63 (19.0)	15 (18.5)
Mother’s age at delivery, y			
<25	30 888 (9.9)	26 (7.8)	5 (6.3)
25-29	94 672 (30.6)	98 (29.5)	25 (30.8)
30-34	111 913 (36.2)	130 (39.2)	25 (30.8)
≥35	72 000 (23.3)	78 (23.5)	26 (32.1)
Mother’s highest educational level, y			
<9	44 528 (14.4)	53 (16.0)	12 (14.8)
10-12	99 503 (32.1)	102 (30.7)	28 (34.6)
≥13	165 442 (53.5)	177 (53.3)	41 (50.6)
Exclusive breastfeeding duration			
Never or <14 d	37 014 (11.9)	45 (13.6)	9 (11.1)
≤2 mo	67 118 (21.7)	82 (24.7)	25 (30.9)
3-5 mo	144 702 (46.8)	143 (43.0)	34 (42.0)
≥6 mo	60 639 (19.6)	62 (18.7)	13 (16.0)

Abbreviation: ALL, acute lymphoblastic leukemia.

^a^ Exact numbers are not presented to blind numbers less than 5 (directly or by calculation through group totals) in accordance with the interpretation of the General Data Protection Regulation by Statistics Denmark.

In the full cohort, 104 132 children (33.6%) were exclusively breastfed for less than 3 months. Among those diagnosed with any cancer, this proportion was 127 (38.3%), and among those diagnosed with ALL, it was 34 (42.0%) (Table 1).

Log-linear trends in HRs for the association of cancer risk with 1 additional month of exclusive breastfeeding are presented in Table 2. While the HR was close to 1 for most of the investigated cancers, log-linear trend adjusted HRs (AHRs) were 0.91 (95% CI, 0.83-0.99) for hematologic cancers overall and 0.90 (95% CI, 0.80-1.01) for BCP-ALL.

**Table 2.** Hazard Ratios of Childhood Cancer Associated With Exclusive Breastfeeding Duration by Childhood Cancer Groups and Diagnoses

Cancer type, breastfeeding duration	Person-years	Events, No.	Crude model		Adjusted model^a^	
			HR (95% CI)	*P* value^b^	Adjusted HR (95% CI)	*P* value^b^
**Any cancer**						
Total	1 679 635	332	NA	NA	NA	NA
Exclusive breastfeeding duration						
Never or <14 d	191 314	45	1 [Reference]	.30	1 [Reference]	.23
≤2 mo	367 242	82	0.95 (0.67-1.38)		0.97 (0.68-1.41)	
3-5 mo	792 367	143	0.77 (0.56-1.09)		0.76 (0.55-1.08)	
≥6 mo	328 713	62	0.82 (0.56-1.20)		0.79 (0.54-1.18)	
Linear trend per 1-mo increase^c^	1 488 321	287	0.98 (0.93-1.03)	.48	0.98 (0.92-1.03)	.34
Exclusive breastfeeding duration, mo						
0-2	558 556	127	1 [Reference]	.06	1 [Reference]	.04
≥3	1 121 079	205	0.81 (0.65-1.01)		0.79 (0.63-0.99)	
**Hematologic cancers**						
Total	NA	124	NA	NA	NA	NA
Exclusive breastfeeding duration						
Never or <14 d	191 390	15	1 [Reference]	.20	1 [Reference]	.13
≤2 mo	367 376	37	1.30 (0.73-2.43)		1.31 (0.73-2.46)	
3-5 mo	792 649	51	0.83 (0.48-1.53)		0.79 (0.45-1.47)	
≥6 mo	328 853	21	0.83 (0.43-1.64)		0.78 (0.40-1.55)	
Linear trend per 1-mo increase^c^	1 488 879	109	0.92 (0.84-1.00)	.055	0.91 (0.83-0.99)	.04
Exclusive breastfeeding duration, mo						
0-2	558 767	52	1 [Reference]	.04	1 [Reference]	.03
≥3	1 121 502	72	0.70 (0.49-0.99)		0.66 (0.46-0.95)	
**ALL**						
Total	NA	81	NA	NA	NA	NA
Exclusive breastfeeding duration						
Never or <14 d	191 402	9	1 [Reference]	.31	1 [Reference]	.24
≤2 mo	367 402	25	1.48 (0.72-3.35)		1.50 (0.73-3.41)	
3-5 mo	792 724	34	0.94 (0.47-2.09)		0.92 (0.45-2.04)	
≥6 mo	328 875	13	0.87 (0.37-2.10)		0.82 (0.35-2.01)	
Linear trend per 1-mo increase^c^	1 489 001	72	0.93 (0.83-1.03)	.15	0.92 (0.82-1.02)	.12
Exclusive breastfeeding duration, mo						
0-2	558 804	34	1 [Reference]	.12	1 [Reference]	.09
≥3	1 121 599	47	0.70 (0.45-1.10)		0.67 (0.43-1.06)	
**BCP-ALL**						
Total	NA	74	NA	NA	NA	NA
Exclusive breastfeeding duration						
Never or <14 d	191 402	8	1 [Reference]	.23	1 [Reference]	.14
≤2 mo	367 407	24	1.60 (0.75-3.81)		1.63 (0.76-3.90)	
3-5 mo	792 742	30	0.94 (0.45-2.20)		0.89 (0.42-2.09)	
≥6 mo	328 877	12	0.91 (0.38-2.31)		0.83 (0.34-2.14)	
Linear trend per 1-mo increase^c^	1 489 026	66	0.91 (0.82-1.02)	.10	0.90 (0.80-1.01)	.07
Exclusive breastfeeding duration, mo						
0-2	558 809	32	1 [Reference]	.09	1 [Reference]	.04
≥3	1 121 619	42	0.67 (0.42-1.07)		0.62 (0.39-0.99)	
**CNS tumors**						
Total	NA	44	NA	NA	NA	NA
Exclusive breastfeeding duration, mo						
0-2	558 907	15	1 [Reference]	.93	1 [Reference]	.91
≥3	1 121 678	29	0.97 (0.53-1.86)		0.96 (0.51-1.88)	
Linear trend per 1-mo increase^c^	1 489 155	39	1.03 (0.89-1.17)	.72	1.03 (0.89-1.18)	.67
**Solid tumors**						
Total	NA	80	NA	NA	NA	NA
Exclusive breastfeeding duration, mo						
0-2	558 854	29	1 [Reference]	.60	1 [Reference]	.58
≥3	1 121 565	51	0.88 (0.56-1.41)		0.87 (0.55-1.41)	
Linear trend per 1-mo increase^c^	1 489 027	66	1.02 (0.92-1.13)	.73	1.01 (0.90-1.12)	.88
**Neuroblastoma**						
Total	NA	14	NA	NA	NA	NA
Exclusive breastfeeding duration, mo						
0-2	558 926	5	1 [Reference]	.89	1 [Reference]	.98
≥3	1 121 716	9	0.92 (0.32-3.00)		0.98 (0.32-3.06)	
Linear trend per 1-mo increase^c^	1 489 213	<14^d^	1.04 (0.81-1.30)	.73	1.05 (0.83-1.34)	.66

Abbreviations: ALL, acute lymphoblastic leukemia; BCP, B-cell precursor; CNS, central nervous system; HR, hazard ratio; NA, not applicable.

^a^ Adjusted for year of birth (linearly), birth weight (linearly, in 1-g intervals), gestational age (linearly, in 1-day intervals), mother’s age at delivery (linearly, in 1-year intervals), mode of birth (vaginal or cesarean delivery), and mother’s highest educational level and stratified by sex and birth order.

^b^ *P* values and 95% CIs were based on the likelihood ratio.

^c^ Children exclusively breastfed for less than 14 days or never were excluded.

^d^ Exact number is not presented to blind numbers less than 5 (directly or by calculation through group totals), in accordance with the interpretation of the General Data Protection Regulation by Statistics Denmark.

In dichotomized analyses, compared with exclusive breastfeeding for shorter periods, exclusive breastfeeding for at least 3 months was associated with a reduced risk of hematologic cancers (AHR, 0.66; 95% CI, 0.46-0.95). This risk reduction was largely attributable to a decreased risk of BCP-ALL (AHR, 0.62; 95% CI, 0.39-0.99). In contrast, risk of CNS tumors (AHR, 0.96; 95% CI, 0.51-1.88), solid tumors (AHR, 0.87; 95% CI, 0.55-1.41), and neuroblastomas (AHR, 0.98; 95% CI, 0.32-3.06) did not vary by exclusive breastfeeding duration (Table 2). The small number of children diagnosed with AML or Hodgkin lymphoma in the cohort precluded estimation of the association of exclusive breastfeeding with these cancers (eTable 1 in Supplement 1). In the supplementary analyses stratified by attained age, birth cohort, and birth mode, we found that children exclusively breastfed for at least 3 months had a decreased risk of BCP-ALL at ages 2 to 6 years and 7 to 14 years (*P* = .58 for interaction), among children born between 2005 and 2011 and children born between 2012 and 2018 (*P* = .20 for interaction), and among children delivered vaginally and those born by cesarean delivery (*P* = .92 for interaction) (eTable 2 in Supplement 1).

## Discussion

In this prospective cohort study, we used unique register data to assess the association between breastfeeding duration and risk of childhood cancer. Our analyses showed that children exclusively breastfed for at least 3 months had a lower risk of BCP-ALL at ages 1 to 14 years than did children breastfed exclusively for less than 3 months or never at all. Also, we observed no association between exclusive breastfeeding duration and risk of CNS tumors or solid tumors at ages 1 to 14 years. Our analyses thereby corroborate previous observations from case-control studies suggesting that breastfeeding is associated with risk of childhood ALL.^6,7,8,9,10,11^ Notably, our results for BCP-ALL align with the approximately 30% reduced ALL risk in children breastfed exclusively for at least 4 months vs never breastfed in recent pooled analyses of international case-control studies including more than 10 000 children with ALL.^11^

Current models posit that childhood BCP-ALL development often begins before birth, when for unknown reasons, an initial genetic event gives rise to a preleukemic clone (such as *ETV6-RUNX1*).^15,16^ The prevalence of preleukemia in neonates is still debated,^28,29^ but most likely, BCP-ALL develops in only a small proportion of children born with preleukemia.^15^ It has long been speculated that in these children, the malignant transformation of a preleukemic clone into ALL is triggered by a dysregulated immune response to infections.^15,16^

The association between breastfeeding and childhood BCP-ALL risk, if determined to be causal, could be mediated by preventing this immunologic dysregulation.^30^ Specifically, breastfeeding provides passive protection against infections and inflammation through antibody transmission and anti-inflammatory properties and also directly influences the shaping of the infant’s gut microbiome, important for immune system maturation.^12,13,14,31^

The role of gut microbiome maturation in childhood ALL pathogenesis and the potential for gut microbiome–targeted preemptive interventions has recently gained increasing attention.^16,30^ Several studies have shown that children with ALL have alterations of their gut microbiome at diagnosis compared with healthy peers.^30,32,33^ While the sequence of events is difficult to disentangle from such studies, murine models of BCP-ALL have found that in genetically predisposed mice (eg, *Pax5*^+/−^), gut microbiome alterations precede leukemia onset.^17,18^ Moreover, in these predisposed mice, leukemia development could be triggered by the destruction of the gut microbiome with antibiotics, even in the absence of infectious stimuli.^17^ It has therefore been suggested that suboptimal enrichment and delayed maturation of the gut microbiome may increase BCP-ALL risk in children born with preleukemic cells.^16,17,30^

In our study, we could not ascertain whether the association between breastfeeding and childhood BCP-ALL risk primarily reflects benefits of breast milk itself, delayed introduction to formula milk, or a combination of both. In the aforementioned international investigation,^11^ introduction to formula milk within the first week of life was associated with an increased risk of childhood ALL even among children who were breastfed for at least 6 months. In the current study, we did not have data to assess the precise age at formula milk introduction. Still, because the Danish definition of exclusive breastfeeding includes supplementation with up to 1 formula meal weekly, our results suggest that breastfeeding is associated with decreased risk of childhood BCP-ALL despite infrequent formula meals.

During the neonatal period, the mode of birth is the predominant factor associated with the composition of the gut microbiota.^34^ Children born by cesarean delivery display reduced overall gut microbiome stability during the first months of life and delayed maturation of the gut microbiome by the second year of life.^31,35^ Furthermore, cesarean delivery is a suspected risk factor for childhood BCP-ALL.^36^ In this view, it is notable that we found longer duration of exclusive breastfeeding to be associated with decreased risk of BCP-ALL similarly across birth modes (ie, both among children born by vaginal delivery and by cesarean delivery). No association between mode of birth and risk of childhood ALL was found in the present study or in previous, larger register-based studies from our group.^37,38^

Some case-control investigations previously showed that longer duration of breastfeeding was associated with decreased risk of rare childhood cancers, including AML,^6,7,11^ Hodgkin lymphoma,^7^ and neuroblastoma.^7,10^ While the limited number of cases in the present study precluded such assessments for AML and Hodgkin lymphoma, our findings did not support an association between exclusive breastfeeding duration and risk of neuroblastoma or solid tumors overall. Moreover, in keeping with our findings, breastfeeding duration was not associated with risk of CNS tumors in a recent pooled analysis of case-control studies including more than 2600 cases^39^ or in previous meta-analyses.^7,10^

### Strengths and Limitations

Strengths of our study include its prospective, register-based design with independent ascertainment of exclusive breastfeeding duration and childhood cancer diagnoses and inclusion of a representative sample of the Danish childhood population across geographic regions and socioeconomic groups. Through linkage between the Danish nationwide registers, we could adjust for child, birth, and family characteristics that have previously been associated with childhood cancer risk and that potentially could confound its association with exclusive breastfeeding.

Among our study’s limitations, we did not have information on exclusive breastfeeding for all children born in Denmark during the study period. This was partly because reporting to the DNCHR became mandatory only in 2012. Therefore, between 2005 and 2011, information on exclusive breastfeeding was available only for 21.2% of the children born in Danish municipalities reporting to the database. However, duration of exclusive breastfeeding was available for only 61.9% of the children born in Denmark between 2012 and 2018, when reporting to the database was mandatory. The relatively high missing percentage in this later period was not confined to certain municipalities and was often due to technical challenges related to electronic data reporting.^24^ Importantly, the observed association between exclusive breastfeeding and childhood BCP-ALL risk pertained to both children born before and children born after reporting became mandatory and also persisted after adjusting for the mother’s educational level in addition to known risk factors for childhood cancer. For these reasons, the missingness pattern was unlikely to have materially affected our statistical inferences and the generalizability of the observations.

Another concern is the potential nondifferential misclassification of exclusive breastfeeding duration, which could bias the presented risk estimates toward the null. Although mothers might misinterpret the term *exclusive breastfeeding*,^40^ our reliance on data collected by health care nurses who were instructed to use a clear definition of exclusive breastfeeding likely minimized such misclassification.^25^ Furthermore, the definition of exclusive breastfeeding used in Denmark differs from that of the World Health Organization, which defines it as the exclusive provision of breast milk without any other food or drink, including water.^41^ This variation in exposure definition should be considered when comparing our findings with those of prior case-control studies. Finally, the morphology code identifying BCP-ALL in the Danish Cancer Register (9835, “precursor cell lymphoblastic leukemia, not otherwise specified”) might also include a few cases of precursor T-cell ALL.

## Conclusions

In this register-based cohort study, we found that longer duration of exclusive breastfeeding (ie, for a period of at least 3 months) was associated with reduced risk of childhood BCP-ALL. This finding is consistent with emerging investigations implicating early gut microbiome maturation in childhood BCP-ALL pathogenesis.^16,17,30^ To inform future preemptive interventions, additional studies should investigate the biologic mechanisms underlying the observed association.

## Notes

Supplement 1. **eTable 1.** Number of Children Aged 1 to 14 Years Diagnosed With Cancer During Follow-Up According to the *International Classification of Childhood Cancer* (Third Edition) **eTable 2.** Hazard Ratios of Childhood BCP-ALL Associated With Exclusive Breastfeeding Duration of 3 Months or Longer Compared With 0 to 2 Months in Strata of Attained Age, Birth Cohort, and Birth Mode

Supplement 2.
Data Sharing Statement
