# Supplementary material for: Exclusive Breastfeeding Duration and Risk of Childhood Cancers
Source: JAMA Netw Open. 2024 Mar 26;7(3):e243115. doi: 10.1001/jamanetworkopen.2024.3115 (PMC10966412; doi:10.1001/jamanetworkopen.2024.3115)
Supplement: Supplement 1. — eTable 1. Number of Children Aged 1 to 14 Years Diagnosed With Cancer During Follow-Up According to the International Classification of Childhood Cancer (Third Edition) eTable 2. Hazard Ratios of Childhood BCP-ALL Associated With Exclusive Breastfeeding Duration of 3 Months or Longer Compared With 0 to 2 Months in Strata of Attained Age, Birth Cohort, and Birth Mode [file jamanetwopen-e243115-s001.pdf]

Supplementary Online Content

Søegaard SH, Andersen MM, Rostgaard K, et al. Exclusive breastfeeding duration and risk of childhood cancers. *JAMA Netw Open*. 2024;7(3):e243115. doi:10.1001/jamanetworkopen.2024.3115

eTable 1. Number of Children Aged 1 to 14 Years Diagnosed With Cancer During Follow-Up According to the *International Classification of Childhood Cancer* (Third Edition)
eTable 2. Hazard Ratios of Childhood BCP-ALL Associated With Exclusive Breastfeeding Duration of 3 Months or Longer Compared With 0 to 2 Months in Strata of Attained Age, Birth Cohort, and Birth Mode

This supplementary material has been provided by the authors to give readers additional information about their work.

© 2024 Søegaard SH et al. *JAMA Network Open*.

eTable 1. Number of children aged 1 to 14 years diagnosed with cancer during follow-up according to the International Classification of Childhood Cancer, Third Edition

	**N**
**Hematologic cancers**	
I. Leukemias, myeloproliferative and myelodysplastic diseases	96
Ia. Lymphoid leukemias	81
Ib. Acute myeloid leukemias	7
Ic-e Other leukemias	8
II. Lymphomas and reticuloendothelial neoplasms	28
IIa: Hodgkin lymphomas	<5[a]
IIb-e: Non-Hodgkin and other lymphomas	<28[a]
**Central nervous system tumors**	
III: CNS and miscellaneous intracranial and intraspinal neoplasms	44
IIIa. Ependymomas and choroid plexus tumor	9
IIIb. Astrocytomas	14
IIIc. Intracranial and intraspinal embryonal tumors	<10[a]
IIId. Other gliomas	<5[a]
IIIe., IIIf., Xa. and Xb. Other CNS tumors	10
**Solid tumors**	
IV. Neuroblastoma and ganglioneuroblastoma	14
V. Retinoblastoma	11
VI. Renal tumors	18
VII: Hepatic tumors	<5[a]
VIII: Malignant bone tumors	<10[a]
IX: Soft tissue and other extraosseous sarcomas	15
Xc-e.: Germ cell tumors and neoplasms of gonads	<5[a]
XI. Other malignant epithelial neoplasms and malignant melanomas	10
**XII. Other and unspecified malignant neoplasms**	84

[a] Exact numbers are not presented to blind numbers under 5 (directly or by calculation through group totals) in accordance with the interpretation of the General Data Protection Regulation by Statistics Denmark.

© 2024 Søegaard SH et al. *JAMA Network Open*.

eTable 2. Hazard ratios of childhood B-cell precursor acute lymphoblastic leukemia associated with exclusive breastfeeding duration ≥3 months compared with 0–2 months in strata of attained age, birth cohort, and birth mode

	N	Person-years	Events	Crude model HR (95% CI)	p for interaction[a]	Adjusted model[b] HR (95% CI)	p for interaction[a]
Exclusive breastfeeding according to attained age					0.57		0.58
2–6 years							
0–2 months	103,580	369,093	24	1.00 (Ref)		1.00 (Ref)	
≥3 months	204,072	719,981	38	0.81 (0.49-1.35)		0.79 (0.47-1.30)	
7–14 years							
0–2 months	40,367	85,887	<5[c]	1.00 (Ref)		1.00 (Ref)	
≥3 months	81,904	196,963	<5[c]	0.45 (0.06-3.18)		0.44 (0.06-3.10)	
Exclusive breastfeeding according to birth cohort					0.27		0.20
2005–2011							
0–2 months	17,298	166,523	10	1.00 (Ref)		1.00 (Ref)	
≥3 months	43,699	417,307	11	0.44 (0.19-1.03)		0.38 (0.16-0.91)	
2012–2018							
0–2 months	86,834	392,286	22	1.00 (Ref)		1.00 (Ref)	
≥3 months	161,642	704,313	31	0.78 (0.45-1.35)		0.74 (0.42-1.29)	
Exclusive breastfeeding according to birth mode					0.99		0.92
Vaginal birth							
0–2 months	78,672	421,937	25	1.00 (Ref)		1.00 (Ref)	
≥3 months	167,825	912,110	35	0.66 (0.40-1.11)		0.61 (0.36-1.03)	
Cesarean section							
0–2 months	25,460	136,872	7	1.00 (Ref)		1.00 (Ref)	
≥3 months	37,516	209,510	7	0.67 (0.23-1.91)		0.65 (0.23-1.85)	

Abbreviations: CI; confidence intervals, HR; hazard ratio.
Confidence limits are based on Wald test due to computational difficulties in handling likelihood ratio in stratified analyses.
[a] P-values for interaction are based on the likelihood ratio.
[b] Adjusted for year of birth (linearly), birthweight (linearly in 1-gram intervals), gestational age (linearly in 1-day intervals), mother's age when giving birth (linearly in 1-year intervals), mode of birth, mother's highest educational level, and stratified by sex and birth order.
[c] Exact numbers are not presented to blind numbers under 5 (directly or by calculation through group totals) in accordance with the interpretation of the General Data Protection Regulation by Statistics Denmark.

© 2024 Søegaard SH et al. *JAMA Network Open*.
