# Supplementary material for: Exclusive Breastfeeding Duration and Risk of Childhood Cancers
Source: JAMA Netw Open. 2024 Mar 26;7(3):e243115. doi: 10.1001/jamanetworkopen.2024.3115 (PMC10966412; doi:10.1001/jamanetworkopen.2024.3115)
Supplement: Supplement 2. — Data Sharing Statement [file jamanetwopen-e243115-s002.pdf]

Data Sharing Statement

Søegaard. Exclusive Breastfeeding Duration and Risk of Childhood Cancers. *JAMA Netw Open*. Published March 26, 2024. doi:10.1001/jamanetworkopen.2024.3115

Data
Data available: No

Additional Information
Explanation for why data not available: According to Danish law, the data material cannot be shared with any external parties without prior permission from data owners and Danish authorities. R code is available from the authors upon reasonable request.
